# Chronic Disease Patterns and Their Relation With Age, Gender, and Number of Visits in Three Primary Care Centers of Riyadh, Saudi Arabia

**DOI:** 10.7759/cureus.30283

**Published:** 2022-10-14

**Authors:** Mohammed A Alsahly, Rakan Ahmed F Al Otaibi, Abdullah I Al Eissa, Muath Owaidh Alilaj

**Affiliations:** 1 Family Medicine, King Abdulaziz Medical City, Riyadh, SAU; 2 Medicine, College of Medicine, King Saud bin Abdulaziz University for Health Sciences, Riyadh, SAU

**Keywords:** multiple visits, kingdom of saudi arabia (ksa), gender role, age and ageing, chronic diseases of comorbidity

## Abstract

Background and objective

Chronic disease is a major health burden and is a leading cause of both morbidity and mortality. However, there is little information regarding this topic in the region of Riyadh, Saudi Arabia. The objective of this study is to assess the pattern of chronic diseases and the role of age, gender, and number of visits in three primary care centers in this region.

Methods

This cross-sectional study was conducted with patients treated at large three primary care centers in Riyadh, Saudi Arabia. The study included all patients who attended one of the three centers within the past four years with one or more chronic diseases, and both genders were included.

Results

There were 700 patients included, of which 437 (62.4%) were female, 263 (37.6%) were male, 327 (31.8%) were diagnosed with type 2 diabetes mellitus (T2DM), and 212 (20.6%) were diagnosed with primary hypertension. There was a significant association between the number of visits and number of diseases and between age and the number of visits. The mean age of patients was 50.7 ± 16.3 years. There was no significant association between gender and the number of diagnoses or number of visits.

Conclusion

This study found a significant relationship between age and the number of visits and number of diseases. T2DM was the most common disease in the population. There was no significant association between gender and the number of diseases or number of visits.

## Introduction

Although comorbidity and multimorbidity may seem similar and are sometimes used interchangeably, they are two distinct terms. Comorbidity was first coined by Feinstein et al.* *(1970) to describe “any distinct additional entity that has existed or may occur during the clinical course of a patient who has the index disease under study” [1]. However, multiple morbidities (MMs) can be defined as “the co-existence of two or more chronic conditions, where one is not necessarily more central than the others” [2]. 

In a study conducted in Qatar in 2020, the prevalence of MM was found to be 22% with a mean number of 2.1 morbidities. The most common patterns of MM in order were hypertension (HTN) and type 2 diabetes mellitus (T2DM), dyslipidemia and T2DM, and dyslipidemia and HTN [3]. In another study from Singapore in 2019, the prevalence of MM was 26%, and the most common patterns were chronic kidney disease (CKD) and HTN, CKD and dyslipidemia, and HTN and dyslipidemia [4]. Other studies have demonstrated a much higher prevalence, like a study conducted in the United Kingdom, which showed a prevalence of 58%, and one in Canada, which showed a prevalence of 53% [5-6].

This issue is a growing challenge for primary healthcare providers, who require a vast body of knowledge and a patient-centered approach to manage such patients. Thus, we believe it is of great importance to try and understand the common patterns of diseases and associated demographics presenting in primary healthcare settings. The aim of this study was to assess the patterns of multimorbidity and characteristics of patients presenting it in primary healthcare clinics in Riyadh, KSA.

## Materials and methods

Study design and settings

This cross-sectional analytical study used chart review to examine patients at three large primary care centers at King Abdul-Aziz Medical City-National Guard (KAMC-NG) in Riyadh, Saudi Arabia. The three primary healthcare centers were the Health Care Specialty Center (HCSC), King Abdul-Aziz City Housing (Iskan Al Yarmouk), and the National Guard Comprehensive Specialized Clinic (NGCSC), which serve around 200,000, 50,000, and 100,000 patients, respectively.

Study population

The study included all patients who attended any of the three primary healthcare centers in King Abdul-Aziz Medical City-National Guard from January 2017 to December 2021 with one or more chronic diseases. Males and females were included.

Data collection

Ethical approval and data were provided by King Abdullah International Medical Research Center (IRB approval number: NRC22R/052/01). Data were extracted from the BESTCare system (ezCaretech Co, Seoul, South, Korea), the digital hospital information system used at King Abdulaziz Medical City, and then entered into and coded using Microsoft Excel (Microsoft Corporation, Redmond, WA, United States). The collected data included patient demographics such as age, sex, and number of visits and the number of diagnosed chronic diseases. Our focus was on hypertension, type 2 diabetes mellitus, hypothyroidism, and other common chronic diseases.

Statistical analysis

**Figure 1:**
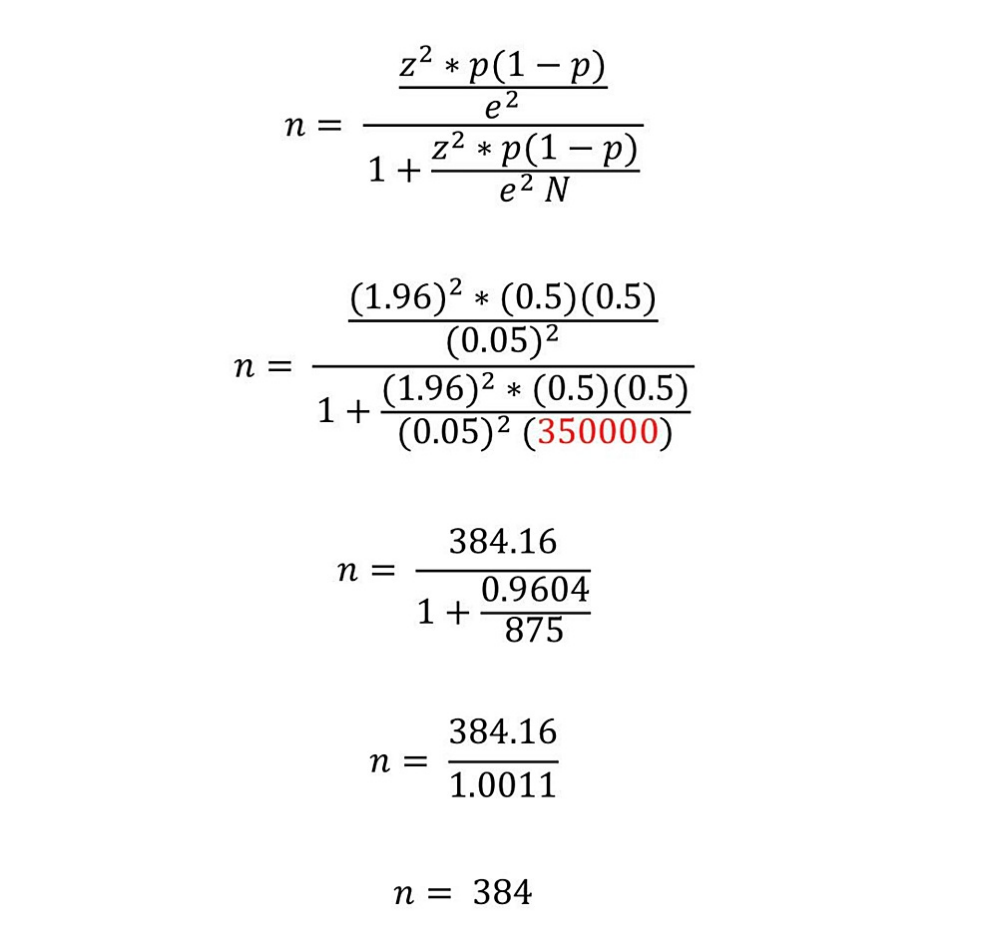
Sample size calculation z: z score for 95% confidence level, p: percentage picking a choice or response, 𝑒: margin of error, N: population size.

The study sample consisted of 700 participants who were randomly selected out of 350,000 patients attending the three primary healthcare centers. The sample size was calculated by the below formula with a minimum number of 384 (Figure 1). The data analysis included two stages. The first stage involved a descriptive analysis where categorical variables were described using frequencies and percentages. The second stage involved a bivariate analysis using a chi-squared test using IBM SPSS Statistics 25.0 (IBM Corp., Armonk, NY). A P-value less than 0.05 was considered statistically significant.

## Results

A total of 700 patients from three centers (HCSC, NGCSC, and Iskan) were involved in the study. Table 1 shows the demographic variables of the sample. Among the patients involved, 45.7% were from HSCS, 31.4% were from NGCSC, and 22.9% were from Iskan. Males accounted for 37.6%, and females accounted for 62.4%.

**Table 1:** Demographics All values presented as numbers and percentages. HCSC: Health Care Specialty Center, NGCSC: National Guard Comprehensive Specialized Clinic, ISKAN: King Abdul-Aziz City Housing (Iskan Al Yarmouk).

Characteristic	n (%)
Hospital name (n=700)	
HCSC	320 (45.7)
NGCSC	220 (31.4)
ISKAN	160 (22.9)
Gender (n=700)	
Male	263 (37.6)
Female	437 (62.4)
Age (n=700)	
18 and less	14 (2.0)
19 to 40	171 (24.4)
41 to 60	324 (46.3)
More than 60	191 (27.3)

**Figure 2:**
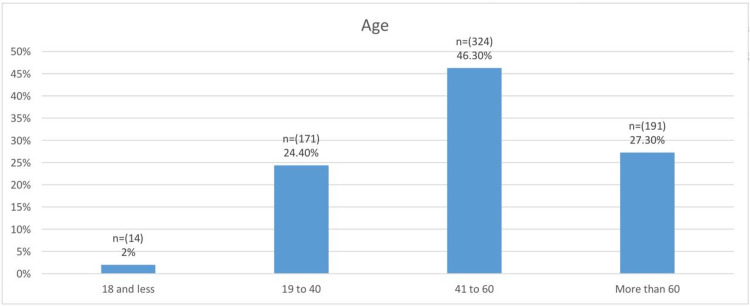
Age group

Figure 2 shows the age groups of the study subjects. There were 14 (2%) subjects who were 18 years old or younger, 171 (24.4%) were between 19 and 40 years, 324 (46.3%) were between 41 and 60 years, and 191 (27.3%) were more than 60 years of age. The mean ages were 48.7 ± 15.2 years for female patients and 53.9 ± 17.5 years for males. The overall mean age was 50.7 ± 16.3 years.

**Figure 3:**
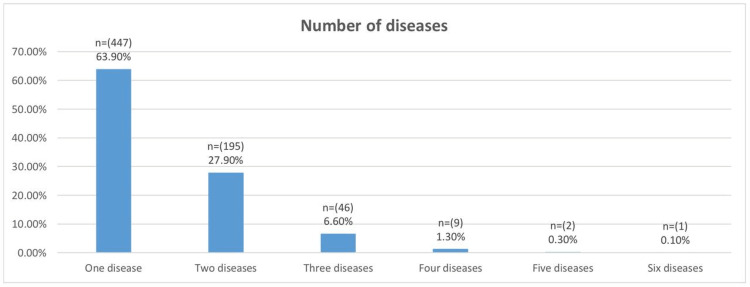
Number of diseases per patient

Figure 3 illustrates the number of diseases per patient. Four hundred forty-seven (63.9%) had one disease, 195 (27%) had two diseases, 46 (6.6%) had three diseases, nine (1.3%) had four diseases, two (0.3%) had five diseases, and one (0.1%) had six diseases. 

Table 2 shows the number of visits per patient and the number of years between making the first and last diagnoses. Out of all study subjects, 386 (55.1%) had one to five visits, 108 (15.4%) had six to 10 visits, 110 (15.7%) had 11 to 20 visits, 50 (7.1%) had between 21 to 30 visits, and 46 (6.6%) had more than 30 visits. As for the time between making the first and last diagnoses, it was less than two years for 629 (89.9%) participants, two to four years for 51 (7.3%), four to six years for 16 (2.3%), six to eight years for two (0.3%), and eight to 10 years for two (0.3%) participants.

**Table 2:** Study factors All values presented as numbers and percentages for categorical.

Factor	n (%)
Number of visits (n=700)	
From 1 to 5 visits	386 (55.1)
From 6 to 10 visits	108 (15.4)
From 11 to 20 visits	110 (15.7)
From 21 to 30 visits	50 (7.1)
More than 30 visits	46 (6.6)
Number of diseases (n=700)	
One disease	447 (63.9)
Two diseases	195 (27.9)
Three diseases	46 (6.6)
Four diseases	9 (1.3)
Five diseases	2 (0.3)
Six diseases	1 (0.1)
Years from first to last diagnoses (n=700)	
From 0 to 2 years	629 (89.9)
From 2 to 4 years	51 (7.3)
From 4 to 6 years	16 (2.3)
From 6 to 8 years	2 (0.3)
From 8 to 10 years	2 (0.3)

Table 3 displays the prevalence of the studied diseases among the sample. T2DM was diagnosed in 327 (31.8%) patients, 212 (20.6%) were diagnosed with primary hypertension, 163 (15.9%) were diagnosed with hypothyroidism, 97 (9.4%) were diagnosed with irritable bowel syndrome, 81 (7.9%) had with asthma, 42 (4.1%) had major depressive disorder, 40 (3.9%) had hyperlipidemia, 25 (2.4%) had gastroesophageal reflux disease, 17 (1.7%) had osteoarthritis, 15 (1.5%) had anxiety disorder, three (0.3%) had chronic kidney disease, two (0.2%) had Alzheimer’s disease, one (0.1%) had chronic obstructive pulmonary disease, one (0.1%) had Parkinson’s disease, and one (0.1%) had ulcerative colitis.

**Table 3:** Study factors All values presented as numbers and percentages for categorical. The total number of patients is 700, and the total number of cases is 1,027.

Disease	Number of patients	Percent	Percent of cases
Type 2 diabetes mellitus	327	31.80%	46.70%
Primary Hypertension	212	20.60%	30.30%
Hypothyroidism	163	15.90%	23.30%
Irritable bowel syndrome	97	9.40%	13.90%
Asthma	81	7.90%	11.60%
Depression	42	4.10%	6.00%
Hyperlipidemia	40	3.90%	5.70%
Gastroesophageal reflux disease	25	2.40%	3.60%
osteoarthritis	17	1.70%	2.40%
Anxiety disorder	15	1.50%	2.10%
Chronic kidney disease	3	0.30%	0.40%
Alzheimer's disease	2	0.20%	0.30%
Chronic obstructive pulmonary disease	1	0.10%	0.10%
Parkinson disease	1	0.10%	0.10%
Ulcerative colitis	1	0.10%	0.10%

Table 4 summarizes the demographic variables, number of diseases per patient, number of visits per patient, and the number of years between making the first and last diagnoses for each center (HCSC, NGCSC, and Iskan).

**Table 4:** Demographics and study factors based on hospital name All values presented as numbers and percentages. HCSC: Health Care Specialty Center, NGCSC: National Guard Comprehensive Specialized Clinic, ISKAN: King Abdul-Aziz City Housing (Iskan Al Yarmouk).

Characteristic	HCSC n (%)	NGCSC n (%)	ISKAN n (%)
Gender (n=700)			
Male	109 (34.1)	88 (40.0)	66 (41.3)
Female	211 (65.9)	132 (60.0)	94 (58.8)
Age (n=700)			
18 and less	5 (1.6)	4 (1.8)	5 (3.1)
19 to 40	77 (24.1)	51 (23.2)	43 (26.9)
41 to 60	149 (46.6)	95 (43.2)	80 (50.0)
More than 60	89 (27.8)	70 (31.8)	32 (20.0)
Number of visits (n=700)			
From 1 to 5 visits	181 (56.6)	99 (45.0)	106 (66.3)
From 6 to 10 visits	49 (15.3)	34 (15.5)	25 (15.6)
From 11 to 20 visits	56 (17.5)	32 (14.5)	22 (13.8)
From 21 to 30 visits	20 (6.3)	25 (11.4)	5 (3.1)
More than 30 visits	14 (4.4)	30 (13.6)	2 (1.3)
Number of diagnoses (n=700)			
One disease	211 (65.9)	125 (56.8)	111 (69.4)
Two diseases	89 (27.8)	72 (32.7)	34 (21.3)
Three diseases	17 (5.3)	17 (7.7)	12 (7.5)
Four diseases	3 (0.9)	4 (1.8)	2 (1.3)
Five diseases	0 (0.0)	1 (0.5)	1 (0.6)
Six diseases	0 (0.0)	1 (0.5)	0 (0.0)
Years from first to last diagnoses (n=700)			
From 0 to 2	286 (89.4)	201 (91.4)	142 (88.8)
From 2 to 4	23 (7.2)	16 (7.3)	12 (7.5)
From 4 to 6	9 (2.8)	2 (0.9)	5 (3.1)
From 6 to 8	1 (0.3)	0 (0.0)	1 (0.6)
From 8 to 10	1 (0.3)	1 (0.5)	0 (0.0)

Table 5 demonstrates the relation between the number of visits and the number of diseases. There was a strong significant association between the two variables (P-value <0.001).

**Table 5:** Test the relation between the number of visits and number of diagnoses *Statistically associated at 0.05 level of significance.

Number of visits/Number of diagnoses n (%)	One disease	Two diseases	Three diseases	Four diseases	Five diseases	Six diseases	P-value
From 1 to 5 visits	332 (74.3)	49 (25.1)	5 (10.9)	0 (0.0)	0 (0.0)	0 (0.0)	<0.001*
From 6 to 10 visits	57 (12.8)	42 (21.5)	9 (19.6)	0 (0.0)	0 (0.0)	0 (0.0)	
From 11 to 20 visits	49 (11.0)	47 (24.1)	12 (26.1)	2 (22.2)	0 (0.0)	0 (0.0)	
From 21 to 30 visits	6 (1.3)	28 (14.4)	13 (28.3)	2 (22.2)	0 (0.0)	1 (100)	
More than 30 visits	3 (0.7)	29 (14.9)	7 (15.2)	5 (55.6)	2 (100)	0 (0.0)	

Table 6 shows the relation between patients’ demographics and the number of visits, which shows significant associations of the number of visits with the center visited (P-value <0.001) and age (P-value <0.001), but no association with gender (P-value = 0.543).

**Table 6:** Test the relation between patients’ demographics and the number of visits *Statistically associated at 0.05 level of significance. HCSC: Health Care Specialty Center, NGCSC: National Guard Comprehensive Specialized Clinic, ISKAN: King Abdul-Aziz City Housing (Iskan Al Yarmouk).

Demographics/Number of visits (per day)	1 to 5 n (%)	6 to 10 n (%)	11 to 20 n (%)	21 to 30 n (%)	More than 30 n (%)	P-value
Hospital name						
HCSC	181 (46.9)	49 (45.4)	56 (50.9)	20 (40.0)	14 (30.4)	<0.001*
NGCSC	99 (25.6)	34 (31.5)	32 (29.1)	25 (50.0)	30 (65.2)	
ISKAN	106 (27.5)	25 (23.1)	22 (20.0)	5 (10.0)	2 (4.3)	
Gender						
Male	149 (38.6)	38 (35.2)	42 (38.2)	14 (28.0)	20 (43.5)	0.543
Female	237 (61.4)	70 (64.8)	68 (61.8)	36 (72.0)	26 (56.5)	
Age						
18 years and less	13 (3.4)	1 (0.9)	0 (0.0)	0 (0.0)	0 (0.0)	<0.001*
From 19 to 40	120 (31.1)	24 (22.2)	22 (20.0)	3 (6.0)	2 (4.3)	
From 41 to 60	164 (42.5)	52 (48.1)	59 (53.6)	29 (58.0)	20 (43.5)	
More than 60	89 (23.1)	31 (28.7)	29 (26.4)	18 (36.0)	24 (52.2)	

Table 7 shows the relation between the demographics and the number of diseases, which shows a significant difference between age groups in the number of diseases (P-value <0.001).

**Table 7:** Test the relation between patients’ demographics and the number of diagnoses *Statistically associated at 0.05 level of significance. HCSC: Health Care Specialty Center, NGCSC: National Guard Comprehensive Specialized Clinic, ISKAN: King Abdul-Aziz City Housing (Iskan Al Yarmouk).

Demographics/Number of diagnoses	One disease n (%)	Two diseases n (%)	Three diseases n (%)	Four diseases n (%)	Five diseases n (%)	Six diseases n (%)	P-value
Hospital name							
HCSC	211 (47.2)	89 (45.6)	17 (37.0)	3 (33.3)	0 (0.0)	0 (0.0)	0.161
NGCSC	125 (28.0)	72 (36.9)	17 (37.0)	4 (44.4)	1 (50.0)	1 (100)	
ISKAN	111 (24.8)	34 (17.4)	12 (26.1)	2 (22.2)	1 (50.0)	0 (0.0)	
Gender							
Male	177 (39.6)	71 (36.4)	14 (30.4)	1 (11.1)	0 (0.0)	0 (0.0)	0.158
Female	270 (60.4)	124 (63.6)	32 (69.6)	8 (88.9)	2 (100)	1 (100)	
Age							
18 years and less	14 (3.1)	0 (0.0)	0 (0.0)	0 (0.0)	0 (0.0)	0 (0.0)	<0.001*
From 19 to 40	142 (31.8)	23 (11.8)	5 (10.9)	1 (11.1)	0 (0.0)	0 (0.0)	
From 41 to 60	195 (43.6)	103 (52.8)	20 (43.5)	3 (33.3)	2 (100)	1 (100)	
More than 60	96 (21.5)	69 (35.4)	21 (45.7)	5 (55.6)	0 (0.0)	0 (0.0)	

## Discussion

The demographic variables in our study are similar to those of other studies that took place in Switzerland and Qatar. In our study, the mean age of participants was 50.7 ± 16.3 years, and females accounted for more than half of the study subjects (62.4% females and 37.6% males). In the Switzerland study, the mean age was 49.6 ± 19.2 years with 52.8% females and 47.2% males, while in the Qatar study, the mean age was slightly lower (43 ± 9.19 years) than that of our sample, with 51.2% females and 48.8% males [3,7].

Among all chronic diseases, T2DM was the most common in our study, followed by primary hypertension. This is consistent with the study conducted in Qatar regarding the prevalence of multimorbidity with T2DM, where primary hypertension was the most common long-term chronic disease [3]. Another study conducted in Brazil found that primary hypertension was the most common, followed by back problems and high cholesterol; T2DM was not among the top five morbidities [8]. The difference in T2DM prevalence between the two studies could be attributed to different genetic backgrounds, lifestyles, and socioeconomic statuses. Regarding this matter, another study conducted in Qatar on the prevalence of non-communicable diseases according to nationality found that T2DM rates were higher among Qataris than among non-Qataris [9].

Research has shown that many demographic and environmental factors, including the increase in the population’s age, have led to an increase in the prevalence of multimorbidity and non-communicable diseases [10]. We found a strong association between age and the number of diagnoses and visits, which is consistent with a cross-sectional study in Scotland, which found that the number of morbidities and multimorbidity increases significantly with age [11]. Another study conducted in Denmark also found that age was significantly associated with the prevalence of multimorbidity [12].

We found no significant difference between males and females in terms of the number of diagnoses and number of visits, but another study found that the number of diagnoses is higher among males than among females. However, this could be attributed to the fact that the studied population consisted mainly of male foreign workers [9]. Other studies conducted in Australia, Germany, and Switzerland support our findings that there is no significant difference between males and females in terms of the number of chronic diseases [7,13-14].

Socioeconomic status, smoking status, level of physical activity, and BMI could have provided more insight into the issue; however, it was unobtainable from the health information system, and this is one of the limitations of our study. Further local studies across a larger population and with more variables such as the ones mentioned earlier are needed to expand our knowledge in regards to multimorbidity in primary healthcare in the Kingdom of Saudi Arabia.

## Conclusions

Our study investigated the patterns of chronic diseases and associated demographic factors in three primary healthcare clinics in Riyadh, Saudi Arabia. We found a significant association between the number of visits and the number of diseases per patient. Additionally, there was a statistically significant difference regarding age in both the number of visits to the clinics and the number of diseases. There was no significant difference regarding gender and the number of diseases.
